# High Self-Control Reduces Risk Preference: The Role of Connectivity Between Right Orbitofrontal Cortex and Right Anterior Cingulate Cortex

**DOI:** 10.3389/fnins.2019.00194

**Published:** 2019-03-11

**Authors:** Mengmeng Wang, Zhiyi Chen, Shunmin Zhang, Ting Xu, Rong Zhang, Tao Suo, Tingyong Feng

**Affiliations:** ^1^School of Education, Institute of Cognition, Brain, and Health, Henan University, Kaifeng, China; ^2^School of Education, Institute of Psychology and Behavior, Henan University, Kaifeng, China; ^3^Faculty of Psychology, Southwest University, Chongqing, China; ^4^Key Laboratory of Cognition and Personality, Ministry of Education, Chongqing, China

**Keywords:** self-control, risk preference, voxel-based morphometry, resting-state functional connectivity, mediation

## Abstract

Risk preference, the preference for risky choices over safe alternatives, has a great impact on many fields, such as physical health, sexual safety and financial decision making. Ample behavioral research has attested that inadequate self-control can give rise to high risk preference. However, little is known about the neural substrates underlying the effect of self-control on risk preference. To address this issue, we combined voxel-based morphometry (VBM) with resting-state functional connectivity (RSFC) analyses to explore the neural basis underlying the effect of self-control on risk preference across two independent samples. In sample 1 (99 participants; 47 males; 20.37 ± 1.63 years), the behavioral results indicated that the scores of self-control were significantly and negatively correlated with risk preference (indexed by gambling rate). The VBM analyses demonstrated that the higher risk preference was correlated with smaller gray matter volumes in right orbitofrontal cortex (rOFC) and right posterior parietal cortex. In the independent sample 2 (80 participants; 33 males; 20.33 ± 1.83 years), the RSFC analyses ascertained that the functional connectivity of rOFC and right anterior cingulate cortex (rACC) was positively associated with risk preference. Furthermore, the mediation analysis identified that self-control mediated the impact of functional connectivity of rOFC-rACC on risk preference. These findings suggest the functional coupling between the rOFC and rACC might account for the association between self-control and risk preference. The present study extends our understanding on the relationship between self-control and risk preference, and reveals possible neural underpinnings underlying this association.

## Introduction

The decisions we make in our daily lives have a pronounced effect on our physical, psychological, and individual or family economics. Specifically, risks and uncertainties pervade our decisions across the lifespan, including physical health (Lusk and Coble, 2005), sexual safety (Harbison et al., 2018), and financial decision making (Engelmann and Tamir, 2009; Angkinand and Wihlborg, 2010; Wen et al., 2014). Notably, risk preference, which is defined as the general tendency to take risks in a particular decision context (Sitkin and Pablo, 1992; Mullins and Forlani, 2005), is a crucial indicator of risk decision making. Individuals with high risk preferences are commonly associated with more maladaptive behaviors, including alcohol consumption, drug abuse, smoking, gambling, and unsafe sexual activity (Donohew et al., 2000; Robbins and Bryan, 2004; Bechara, 2005; Bickel et al., 2012). Prior literature has indicated that risk preference results from a lack of self-control (Freeman and Muraven, 2010; Ryan et al., 2013). Although the relationship between self-control and risk preference has been explored in the behavioral field, little is known about the neural substrates underlying the effects of self-control on risk preference.

A large number of human decisions involving a balance between anticipated rewards and risks are regulated by self-control, which can resist immediate temptations in favor of long-term goals (Kool et al., 2013). The dual-system model, a more systematic explanation for risk behavior, provides a reliable explanation for the impact of self-control on risk preference. According to this theory, risk behavior is recognized as the result of competition between the instinctive affective system and the controlled deliberative system (Casey et al., 2008; Steinberg, 2008). The instinctive affective system, also known as the “hot” system, is spontaneous and automatic, relying upon affective input, such as the expectation of reward following risk behavior (Galvan et al., 2006, 2010). Notwithstanding this, the controlled deliberative system (the “cool” system) is characterized as involving more purely cognitive processes. The affective system can be easily triggered, for example by affectively pursuing rewards due to high risk preference. Meanwhile, the controlled system could block these affective impulses and facilitate deliberative decision making (Cohen, 2005; Knoch and Fehr, 2010). Self-control has been quintessentially deemed a part of the controlled deliberative system (Kruglanski, 2018). This system underlies goal-directed behavior and requires a volitional control or willpower to be effective (Metcalfe and Mischel, 1999). However, the avoidance of risk preference requires effective self-control (de Ridder et al., 2012), which is initiated by inner responses and undesired behavioral tendencies (Tangney et al., 2004; Carver and Scheier, 2012). According to the process model of self-control depletion, self-control failure is caused by the motivated switching to “want-go” goals, which are carried out for personal enjoyment and gratification, such as reward seeking (Inzlicht et al., 2014). Taken together, risk behaviors are performed because the motivation to rewards cannot be resisted by low cognitive control ability. Accordingly, it is presumable that the relative strength of the reward evaluation system compared to the cognitive control system should be highlighted as a core component of the association between self-control and risk preference.

The dual system model comprises of two distinct neurobiological sub-systems: the “cognitive control” system, which mainly involves the lateral prefrontal cortex (lPFC), parietal cortices (PC), anterior cingulate cortex (ACC) (Steinberg, 2008), and the “socioemotional” system, which is located in the limbic and paralimbic areas of the brain, such as the ventral striatum and orbitofrontal cortex (OFC). Specifically, in the “cognitive control” system, the lPFC is closely linked to deliberative processing and self-control in the suppression of affective impulses (Mcclure and Bickel, 2015; Su et al., 2018). The lPFC implements control in part by biasing processing through the connection with the posterior parietal cortex (PPC). The activation of the PPC is associated with attending to and evaluating the risks involved in decision making (Huettel et al., 2005; Christopoulos et al., 2009). The lPFC, which is responsible for executive cognitive control, might also collaborate with regions implicated in conflict monitoring, such as the ACC (Han et al., 2012; Gowin et al., 2013). In the “socioemotional” system, this striatum is believed to encode the communication between expected value and received rewards in risk decisions (Breiter et al., 2001; Tobler et al., 2009; Wang et al., 2015). Neural activity in the OFC encodes possible rewards by integrating the history of the latest outcome into expected outcomes (Elliot, 1999; Ernst et al., 2002; Akitsuki et al., 2003). Meanwhile, the hyperactivity of the OFC has been observed when individuals were engaged in risk-taking behavior (Jentsch et al., 2010). In some pathological studies, the OFC has been found to be activated in obsessive compulsive disorders (Breiter et al., 1996; Mataix-Cols et al., 2004) and in cocaine abusers (Arana et al., 2003); this region is involved with processing the reward values of stimuli and motivating behavioral responding to rewards (Goldstein et al., 2007). The OFC, once activated, needs cognitive control to allow individuals to, for example, not take a drug (Volkow and Fowler, 2000), but individuals with drug addictions have disrupted self-control (Baler and Volkow, 2006). Therefore, urges for reward caused the individuals with low self-control to not be able to block this motivation to rewards. As a result, harmful consequences and risk behaviors resulted (Evans et al., 2003). As alluded to earlier, the high risk preference was induced by ineffectual self-control at the cognitive level, underlying intense motivation for reward. Correspondingly, individuals with low self-control, which means weak “cognitive control,” had high tendencies toward risk behavior, owing to relative hyperactivity of the “socioemotional” system (Steinberg, 2008). Taken together, we postulated that the brain regions in the control network (i.e., the ACC and the prefrontal cortex) and “socioemotional” system (i.e., the OFC) could account for the relationship between self-control and risk preference.

In the present study, we sought to explore the neural substrates underlying the association between self-control and risk preference using voxel-based morphometry (VBM), in conjunction with the resting-state functional connectivity (RSFC) methods. Previous work has used this method to explore the brain morphometric and rs-fMRI functional connectivity in various brain disorders (Liao et al., 2011a,b). To our knowledge, there are notably few studies that have investigated the relationship between self-control and risk preference, based on combination of this two methods. The VBM can characterize the anatomical trait throughout the brain (Ashburner and Friston, 2000; Bellgrove et al., 2004). This method is also suitable to detect individual differences in cognitive control and personality (Spampinato et al., 2009). The RSFC is a technique used to explore the intrinsic functional architecture of the human brain, when subjects were not engaged in external tasks (Lewandowska et al., 2008). Intriguingly, it has been widely acknowledged that altered gray matter volumes (GMV) in some regions was ordinarily accompanied by corresponding changes of RSFCs (Gili et al., 2011). Whilst combining these two methods can drastically broaden our knowledge about the neural correlates of some behavior (Li et al., 2012). Therefore, the Self-control Scale (SCS) and the Wheel of Fortune task (WOF) were used to assess individuals’ ability of self-control and risk preference, respectively. In sample 1, we performed whole-brain VBM analyses to search the GMV of the regions correlated with risk preference. In sample 2, we defined the brain regions in which the GMV had exhibited significant correlation with risk preference in sample 1 as seed regions to calculate voxel-wise functional connectivity. The functional connectivity related with risk preference and self-control were, respectively examined. Finally, mediation analysis was performed to further explore whether RSFCs contributed to the relationship between self-control and risk preference or not.

## Materials and Methods

### Participants and Procedure

Ninety nine healthy college students were recruited as the sample 1 (47 males; age, 20.37 ± 1.63 years), whilst 90 healthy college students were enrolled as the independent sample 2. Ten participants were excluded due to the excessive head movement [exceed framewise displacement (FD) > 0.2 mm] during scanning (see details below), and 80 participants remained in sample 2 (33 males; age, 20.33 ± 1.83 years). Each participant was right-handed and had normal or corrected-to-normal vision. No history of psychiatric disorder was reported as well. All participants gave the informed consent. Prior to MRI scanning, participants were required to finish the self-control scale and the WOF. Afterward, they were reimbursed for participation.

### Measures

#### The Self-Control Scale

Self-control ability was measured by the SCS, which has been widely considered to be able to assess the multiple domains of individual self-control (Tangney et al., 2004). Specifically, 36 items are included in this scale and can be divided into 5 dimensions, including general self-discipline (11 items), impulsive control (10 items), healthy habit (7 items), work/study ethic (4 items), and reliability (4 items). Each item is rated on the rank from 1 (strongly disagree) to 5 (strongly agree) (e.g., “I am good at resisting temptation”). The score of negative wording items was reversed so that the higher total score can represent the better self-control ability. Self-control scores in the present study were normally distributed (Kolmogorov-Smirnov *z* = 0.810, *p* = 0.528). This scale showed a good internal consistency (α = 0.89) (Tangney et al., 2004).

#### The Wheel of Fortune Task

**FIGURE 1:**
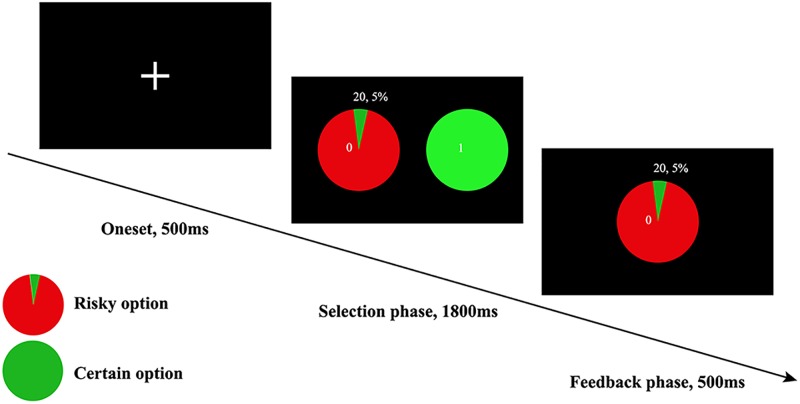
The WOF task, only the winning version was displayed here, which meant when an individual chose to accept the risky choice, then he or she would get ¥ 20 as feedback. The trial-by-trial feedback was real in accordance to the probabilities presented, and participants’ payment was based on their performance on all trials.

In this study, the risk preference was assessed via the WOF (Ernst et al., 2004), in which participants indicated their preference between a certain option and a risk option for monetary rewards. The certain options were rewarded with fixed ¥ 1, and the risk options comprised combinations of 19 probabilities (5 to 95% with an interval of 5%) and 9 monetary amounts (¥ 1 to 9; with an interval of ¥1; averaged ¥ 5), yeilding a total of 171 trials (19 probabilities × 9 monetary amounts) in one session. During each trial, if risky option was chosen, a blue dot would stop either in green (indicating win) or red area (indicating they earned nothing) of the wheel as a feedback. Alternatively, if the certain option was chosen, the participant would surely acquire the fixed rewards (see Figure 1). Each subject was informed that their payment would be the monetary payment they got during the whole task. Of note, the expected value of rewards can affect individuals’ choice. Therefore, the expected value of rewards in the fixed option and risky option were equivalently designed (Kahneman and Tversky, 2013). In line with previous study, the gambling rate was obtained from the ratio of risky choice selected in all trials. Ultimately, the gambling rate was used to measure risk preference (Ernst et al., 2004). The higher gambling rate indicates higher risk tolerance.

### fMRI Acquisition

The structural MRI and resting-state fMRI scans were obtained with a Siemens 3T scanner (Siemens MAGNETOM Trio TIM, Erlangen, Germany). The magnetization prepared rapid acquisition gradient-echo (MPRAGE) sequence (128 slices; TR = 2530 ms; TE = 3.39 ms; flip angle = 7°; 256 × 256 matrix) was used to acquire high-resolution T1-weighted anatomical images (voxel size = 1 mm^3^ × 1 mm^3^ × 1.33 mm^3^). Resting-state fMRI images were acquired using T2^∗^-weighted Echo Planar Imaging (EPI) sequence (TR = 2000 ms, TE = 30 ms, flip angle = 90°, resolution matrix = 64 × 64, FOV = 200 mm^2^ × 200 mm^2^, 33 slices, voxel size = 3.1 mm × 3.1 mm × 3.6 mm). Participants were required to keep their eyes closed without sleeping and thinking during the scan. These scans lasted for 8 min, and incorporated 240 volumes.

### Date Analyses

#### VBM Analyses

Voxel-based morphometry analyses were implemented via SPM12, in conjunction with DARTEL and vbm8 toolbox^1^.

##### Preprocessing

Before VBM analyses, the SPM12 was used to display each MRI image for checking whether there were artifacts and gross anatomical abnormalities. As indicated by previous guidance (Ashburner, 2007), all these structural images were manually adjusted to make the anterior commissar match the origin (0, 0, 0), and 3-dimensional Montreal Neurological Institute (MNI) space primarily. Then, these reoriented images were segmented into gray matter, white matter and cerebrospinal fluid. Afterward, the versions of the gray and white matter tissues imported by DARTEL were used to generate the flow fields and a series of template images. Subsequently, those obtained images were smoothed with 8 mm Gaussian FWHM, modulated, and spatially normalized to create Jacobian scaled GM images resliced to 2 mm × 2 mm × 2 mm voxel size in MNI space.

##### Second level modeling analyses

The multiple linear regression was performed to capture the brain regions that were correlated with risk preference in sample 1 (99 participants). In this model, the gambling rate was defined as a covariate of interest, whilst the age and gender were included as covariates of no interest according to previous findings (Kulynych et al., 1994; Good et al., 2001). Afterward, the mask with absolute threshold of 0.2 was performed to restrict the gray matter areas (Guo et al., 2017). Then, the MATLAB script “get_totals”^2^ was used to extract the regional GMV. Eventually, T contrasts were applied to explore the voxels that correlated with risk preference with a threshold at *p* < 0.001. The final results were corrected by small volume correction (Sphere at peak MNI; radius of VOI = 20 mm).

#### RSFC Analyses

##### Preprocessing

The rs-fMRI images were preprocessed in the DPARSF toolbox^3^ (Yan and Zang, 2010). Firstly, to preserve from the distortion magnetization disequilibrium and the participant’s adaptation to the scanning noise, the first 10 volumes of each participant were discarded. The remaining 230 volumes needed to be corrected for temporal shifts between slices and correcting for motion. Following this, all realigned images were normalized to the MNI template in 3 mm^3^ × 3 mm^3^ × 3 mm^3^, and smoothed with an isotropic 4 mm FWHM Gaussian kernel. To reduce the impact of head movement and nuisance signals, the white matter signal, cerebrospinal fluid signal (CFS), global signal, and head motion data were regressed out (Birn et al., 2006; Auer, 2008; Fox et al., 2009). Then, the temporal filtering (0.01–0.08 Hz) and detrending were performed to obtain low-frequency fluctuation from resting state fMRI data. Given the motion-related signal in resting-state fMRI data cannot be fully removed by regression of motion estimates (Power et al., 2012; Yan et al., 2013), the frame-wise motion censoring was performed. The threshold of FD > 0.2 mm as well as 1 back and 2 forward neighbors were used to remove volumes (Power et al., 2013). Motion censoring may result in a large number of eliminated volumes and too few remaining volumes that can lead to unreliable results. On this account, a 5-min criterion was set. Accordingly, 10 participants who had less than 5 min data remaining after censoring were excluded (Power et al., 2013). The mean FD for each participant was further regressed out at the group-level analyses.

##### Functional connectivity analyses

In functional connectivity analyses, the right orbitofrontal cortex (rOFC) derived from the outcomes in sample 1 were identified as seed region to calculate the whole-brain functional connectivity maps in the independent sample 2. Similarly, The functional connectivity was calculated in sample 2 based on seed region of the right posterior parietal cortex (rPPC). In order to produce functional connectivity maps, the BOLD time course from seed regions were extracted, and correlation of time course between each seed regions and the BOLD of all other brain voxels were computed. Then, all maps were transformed with the Fisher *z*-values. To further determine the relations between functional connectivity of seed regions and risk preference, we performed the correlation analyses between this *z*-valued functional connectivity maps and individual’s gambling rate. The regions from correlation analyses (*p* < 0.05, AlphaSim correction, and cluster size ≥187) were saved as ROIs for subsequent analyses. Afterward, to explore the relationship between self-control and this functional connectivity correlated with risk preference, we calculated the correlations between self-control and the connectivity values which were extracted from the seed regions’ connectivity map of each participant. Finally, the mediation analysis was performed to explore the influence of this functional connectivity on the effect of self-control on risk preference.

## Results

### Behavioral Results

**FIGURE 2:**
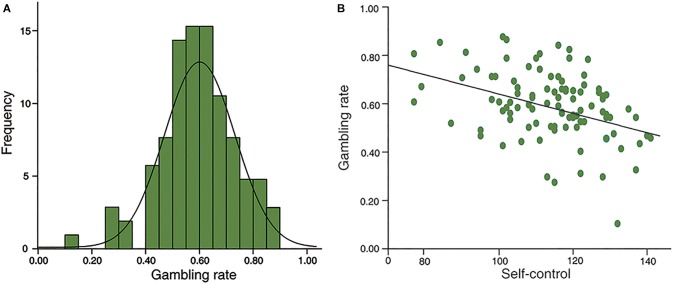
Behavioral results. **(A)**, the distributions of risk preference in sample 1; **(B)**, self-control was negatively correlated with risk preference significantly (*r* = –0.397, *p* < 0.001).

The distribution is normal in self-control (Kolmogorov-Smirnov *z* = 0.810, *p* = 0.528) and gambling rate (Smirnov *z* = 0.588, *p* = 0.880; see Figure 2A). Furthermore, no gender difference was found in self-control (*t* = 1.567, *df* = 97, *p* = 0.120), or in gambling rate (*t* = 1.115, *df* = 97, *p* = 0.268). In addition, there were no significant correlations between age and self-control (*r* = 0.174, *p* = 0.086), or gambling rate (*r* = -0.107, *p* = 0.293). Moreover, in line with previous studies, self-control scores were negatively associated with gambling rate (*r* = -0.397, *p* < 0.001; Figure 2B).

### Neuroanatomical Correlates of Risk Preference

**FIGURE 3:**
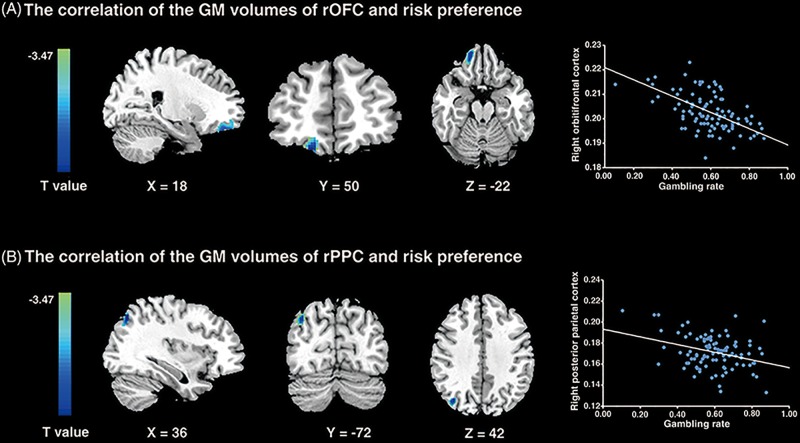
VBM results. **(A)** The gambling rates were negatively correlated with the GM volumes in the right orbitofrontal cortex (*p* < 0.001; small volume corrected). **(B)** The similar correlation was found between risk preference and the GM volumes of the right posterior parietal cortex (*p* < 0.001; small volume corrected). The scatter plots on the right are presented for visualization and not for statistical inference.

To examine the neural substrates underlying risk preference, whole-brain VBM analyses were performed in sample 1. The results demonstrated that the GMV in rOFC (MNI peak coordinates: 18, 50, -22; voxels = 346; *p* < 0.001, small volume correction; see Figure 3A and Table 1) and the rPPC (MNI peak coordinates: 36, -72, 42; voxels = 119; *p* < 0.001, small volume correction; see Figure 3B and Table 1) were negatively associated with gambling rate. These results suggested that the GMV of rOFC and rPPC might be the underlying neuroanatomical basis of risk preference.

**Table 1:** Areas of brain structures significantly correlated with risk preference only -: the brain regions negatively correlated with risk preference (*p* < 0.001; corrected).

Brain regions	MNI	Voxels	T
Only -			
R. orbitofrontal cortex	18, 50, -22	346	-4.18
R. posterior parietal lobe	36, -72, 42	119	-3.43


### RSFC Results

**FIGURE 4:**
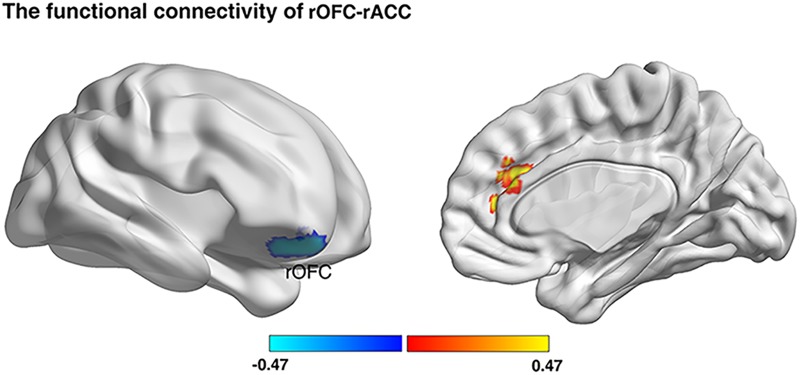
Resting-state functional connectivity result. We defined the rOFC and the rPPC bases on the VBM analyses, respectively, as masks. Functional connectivity between seed regions and rACC was positively correlated with gambling rates (*p* < 0.05; Alphasim corrected, cluster size >187).

Previous studies have demonstrated that the altered GMV in brain regions were accompanied by the altered functional coupling between these altered regions with other related regions (Lui et al., 2009; Gili et al., 2011). Consequently, we firstly investigated whether risk preference would be predicted by functional connectivity with regions (rOFC, MNI: 18, 50, -22; rPPC, MNI: 36, -72, 42) from the VBM results. The results showed that the gambling rate was positively correlated with functional connectivity between rOFC and right anterior cingulate cortex (rACC) under AlphaSim correction (see Figure 4 and Table 2). Notably, no other functional connectivity had a significant correlation with risk preference with the seed region of rPPC. Then, a correlational analysis was conducted to examine the relationship between self-control and functional connectivity of rOFC-rACC. The result demonstrated that the functional connectivity of rOFC-rACC was negatively correlated with self-control scores (*r* = -0.320, *p* = 0.004).

**Table 2:** Functional connectivity correlated with risk preference (*p* < 0.05, Alphasim corrected; rOFC, cluster size >187).

Seed	Region	BA	Voxels	MNI	Correlation coefficient
R. orbitofrontal cortex	R. anterior cingulate cortex	32	320	15, 33, 9	0.327


### The Mediation Analysis

**FIGURE 5:**
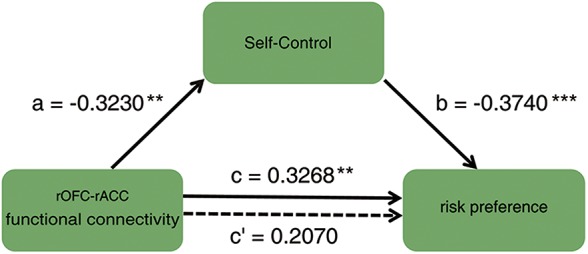
Mediation result: the association between the functional connectivity of rOFC-rACC and risk preference was completely mediated by self-control; ^∗^*p* < 0.05; ^∗∗^*p* < 0.01; ^∗∗∗^*p* < 0.001.

To investigate how the functional connectivity of rOFC-rACC contributed to the effect of self-control on risk preference, mediation analysis using the INDIRECT procedure (Preacher and Hayes, 2008; Hayes and Scharkow, 2013) with 5000 bootstrap samples in SPSS (Statistical Product and Service Solutions) was performed. The estimate of the mediated effect a^∗^b/c = 0.370, and the 95% confidence for intervals of 0.031 and 0.235, suggesting that 37% of the prediction of risk preference by functional connection of rOFC-rACC may have been mediated by self-control (see Figure 5). This result indicated that the functional coupling of the rOFC-rACC might be crucially responsible for the effect of self-control on risk preference.

## Discussion

The present study investigated the neural substrates underlying the effect of self-control on risk preference by combining the VBM and RSFC analyses. In line with previous behavioral studies, the score of self-control was negatively associated with risk preference. The VBM results showed that the GMV of both rOFC and rPPC were negatively correlated with gambling rate. Moreover, increased connectivity between rOFC and rACC was positively correlated with more gambling rate. Finally, the mediation analysis revealed that self-control robustly mediated the effect of functional connectivity of rOFC-rACC on risk preference. Taken together, those findings provide a novel insight into the neural substrates accounting for the association between self-control and risk preference, and extend our understanding on risk preference.

In line with previous research, the scores of self-control were negatively correlated with gambling rate. A considerable number of studies have found that inadequate self-control can result in more maladaptive behaviors, such as addiction (Bechara, 2005; Fox et al., 2008; Bickel et al., 2012), impulsive buying (Rose, 2007; Vohs and Faber, 2007). Furthermore, straightforward studies on the relationship between self-control and risk preference also found that lacking of self-control could lead to more risky activities (Wulfert et al., 1999; Martins et al., 2004; Freeman and Muraven, 2010; Friehe and Schildberg-Hörisch, 2014). Therefore, in accordance with prior observations, our behavioral results revealed an intimate relationship between self-control and risk preference.

The whole-brain VBM analyses demonstrated that the GMV of the rOFC and rPPC were inversely correlated with risk preference, indicating that individuals with less GMV in these regions might have increased the preference for risk. In general, quite a few studies have found that lesions in the parietal cortex impede real-time updating of the probability of winning in gambling. Therefore, this financially challenged adjustment might lead to the experience of financial loss (Studer et al., 2015). Functional neuroimaging studies have highlighted that elevated activation of the parietal cortex assesses high risk in decision making (Symmonds et al., 2011; Studer et al., 2013). More remarkably, one robust study proved that the GMV of rPPC can predict individuals’ risk preference (Gilaie-Dotan et al., 2014). Thus, these studies demonstrably underscored that the parietal cortex made a critical contribution to risk preference. It is worthwhile to note that the OFC was involved in risky decision-making (Fukui et al., 2005; Jentsch et al., 2010). Increasing uncertainty of choice, which is ordinarily involved in risk, could induce the activation of OFC (Critchley et al., 2001). Specifically, the evaluation of the value of rewards in risk decision-making was consistently mediated by activation of the OFC (Ernst et al., 2002; Akitsuki et al., 2003). Additionally, it was confirmed that less GMV of the OFC was associated with more risky behaviors (Matsuo et al., 2009; Peper et al., 2013). These studies suggested that the OFC played a critical role in reward evaluation during risk decision making. The VBM method can predict individual differences in cognitive processes by comparing a voxel-wise of GMV, or neuronal density, and has been increasingly applied in pathological research (Cousijn et al., 2012). Previous evidence has suggested that reduced GMV might be expressed by reduced neuron density, which impedes the transmission efficiency of cognitive processes (Kanai and Rees, 2011). Accordingly, we interpret the decrease in GMV of PPC and OFC as limiting the comprehensive evaluation of risk levels and rewards, due to the inefficient information process. In brief, the rOFC and rPPC could be considered as the neuroanatomical substrates of risk preference.

In order to examine the functional neural substrates of the relationship between self-control and risk preference, the functional connectivity analyses were conducted. The results indicated that the enhanced coupling of the rOFC and rACC was positively correlated with the gambling rate. Many studies have provided convincing evidence that increased rACC activity was related to a lower probability of options in decision making (Elliot, 1999; Koechlin et al., 2000; Volz et al., 2003, 2005) or uncertain conditions that were generally contained in risk choice (Rao and Davim, 2008; Hewig et al., 2009; Payzan-LeNestour et al., 2013). Moreover, the risky choices were accompanied by increased activity in the ACC, which monitored the potential conflicts (Blair et al., 2006). Furthermore, some pathological studies found that patients with OFC damage were insensitive to differing risk conditions (Bechara et al., 2000; Hsu et al., 2005). Specifically, the OFC was widely observed to be involved in processing the subjective value of rewards, suggesting that there is a close relationship between the OFC and risk preference (Levy and Glimcher, 2011; Lin et al., 2012). More importantly, there were the greater activations of the ACC and OFC exhibited when individuals were making relatively high-risk decisions (Ernst et al., 2004; Rogers et al., 2004). It is paramount to note that elevated activation in the ACC is associated with tension between reward seeking and loss avoidance. In other words, the elevated activity in the ACC can be viewed as a state of conflict (Paulus et al., 2001), resulted from the preference for reward under high-risk circumstances (Rao and Davim, 2008; Hewig et al., 2009). Considering the above, the OFC and ACC may serve reward seeking and conflict monitoring functions. The functional connectivity between the rOFC and rACC might signal the pursuit of larger reinforcers, which can result in hyperactivation of the “socioemotional” system. Nevertheless, activation of the rACC in the “cognitive control” system only monitors conflict in decision making but cannot successfully override hyperactivation of “socioemotional” system. Consequently, the enhanced functional connectivity of the rOFC-rACC may be involved in the intense conflict of reward seeking, which gives rise to high risk preference. The RSFC is essential for individuals with high risk preferences. Collectively, our RSFC results indicated that the functional connectivity of the rOFC-rACC might be the neural representation of risk preference.

Importantly, the mediation analysis identified that the impact of the rOFC-rACC functional connectivity on risk preference was robustly mediated by self-control. Notably, it has been alluded that the OFC evaluated rewards in risky decision making (Ernst et al., 2002; Akitsuki et al., 2003), and that the rACC monitored the state of conflict construed by reward seeking and loss avoidance (Carter et al., 1998; Botvinick et al., 2004; Ridderinkhof et al., 2004; Blair et al., 2006). These findings indicated that the functional connectivity of rOFC-rACC was a signal of pursuing high-value rewards. Moreover, high sensitivity for rewards showed an inclination to high risk preference (Leiserson and Pihl, 2007). However, in favor of loss avoidance, strong self-control ability can restrain the desire for obtaining rewards, which triggers activation of the “socioemotional” system. Such a restraint might restrain reward seeking elicited by the activation of the OFC and ACC (Jimura et al., 2013). In contrast, low self-control, or the relatively weak activity of the “cognitive control” system, led to an inability to resist irrational reward seeking. Accordingly, heightened activity of the “socioemotional” system compelled individuals toward risky activity (Steinberg et al., 2015). In brief, the clue of monetary reward activated the OFC and ACC. Once these brain regions are activated, it would motivate individuals to pursuit a reward that might result in a harmful consequence and self-control failure. At this point, the more adequate self-control will be needed to resist this motivation which may cause risk reference. Therefore, risk behaviors was the fruit of the failure of self-control induced by reward purchasing. Taken together, the functional connectivity of rOFC-rACC might present the neural substrates responsible for the relationship between self-control and risk preference.

However, the present study inevitably has some limitations. Primarily, the self-control ability was assessed by a scale, which is less effective than a behavioral task. It is advocated that a behavioral task can be used to estimate the self-control ability under sufficient conditions. It is noteworthy that trait self-control is relatively malleable (Bergen et al., 2012). In the future, it would shed light on holding definite promise for ameliorating the problem of risky behavior, if a reliable way can be found to help individuals with high risk preference to increase their self-control strength. As a second limitation, this study is just a correlative study, which is not able to draw a causal conclusion. Thus, future research can use more experimental methods, such as task fMRI method, to explore the causal effect of self-control on risk preference in depth.

In conclusion, the present investigation showed that self-control was negatively associated with risk preference, suggesting that inadequate self-control ability is associated with higher risk preference. Furthermore, the VBM analyses indicated that the GMV of the rOFC and rPPC were negatively correlated with risk preference. These results demonstrated that the rOFC and rPPC might be the neuroanatomical correlations of risk preference. Moreover, the RSFC results indicated that the functional connectivity of rOFC-rACC was positively correlated with risk preference. We further identified the completely mediating role of self-control in the relationship between these functional connectivity and risk preference. Overall, our study provides a novel insight into the neural substrates underlying the relationship between self-control and risk preference, and extends our understanding on risk preference.

## Data Availability

The datasets for this manuscript are not publicly available because the datasets the privacy information of the subjects, and a confidentiality agreement has been signed to protect this. Requests to access the data sets should be directed to the corresponding author, TF, fengty0@swu.edu.cn.

## Ethics Statement

This study was carried out in accordance with the recommendations of Southwest University institutional review board with written informed consent from all subjects. All subjects gave written informed consent in accordance with the Declaration of Helsinki. The protocol was approved by the Southwest University Institutional Review Board.

## Author Contributions

MW, ZC, SZ, RZ, and TX designed the study and collected and analyzed the data. MW, TS, and TF wrote the manuscript. TF and TS revised the manuscript. All authors approved the version to be published.

## Conflict of Interest Statement

The authors declare that the research was conducted in the absence of any commercial or financial relationships that could be construed as a potential conflict of interest.

## Footnotes

1 http://www.fil.ion.ucl.ac.uk/spm/software/spm12/

2 http://www.cs.ucl.ac.uk/staff/g.ridgway/vbm/get_totals.m

3 http://rfmri.org/dpabi
